# Insights from HuR biology point to potential improvement for second-line ovarian cancer therapy

**DOI:** 10.18632/oncotarget.7840

**Published:** 2016-03-02

**Authors:** Yu-Hung Huang, Weidan Peng, Narumi Furuuchi, James B. DuHadaway, Masaya Jimbo, Andrea Pirritano, Charles J. Dunton, Gary S. Daum, Benjamin E. Leiby, Jonathan R. Brody, Janet A. Sawicki

**Affiliations:** ^1^ Lankenau Institute for Medical Research, Wynnewood, PA 19086, USA; ^2^ Department of Biochemistry and Molecular Biology, Drexel University College of Medicine, Philadelphia, PA 19129, USA; ^3^ Main Line Gynecologic Oncology, Lankenau Medical Center, Wynnewood, PA 19096, USA; ^4^ Main Line Health Laboratories, Lankenau Medical Center, Wynnewood, PA 19096, USA; ^5^ Division of Biostatistics, Thomas Jefferson University, Philadelphia, PA 19107, USA; ^6^ Department of Surgery, Thomas Jefferson University, Philadelphia, PA 19107, USA; ^7^ Sidney Kimmel Cancer Center, Thomas Jefferson University, Philadelphia, PA 19107, USA

**Keywords:** HuR, ovarian cancer, gemcitabine, carboplatin, WEE1

## Abstract

This retrospective study aimed to investigate the role that an RNA-binding protein, HuR, plays in the response of high-grade serous ovarian tumors to chemotherapeutics. We immunohistochemically stained sections of 31 surgically-debulked chemo-naïve ovarian tumors for HuR and scored the degree of HuR cytoplasmic staining. We found no correlation between HuR intracellular localization in tumor sections and progression free survival (PFS) of these patients, 29 of whom underwent second-line gemcitabine/platin combination therapy for recurrent disease. Ribonucleoprotein immunoprecipitation (RNP-IP) analysis of ovarian cancer cells in culture showed that cytoplasmic HuR increases deoxycytidine kinase (dCK), a metabolic enzyme that activates gemcitabine. The effects of carboplatin treatment on HuR and WEE1 (a mitotic inhibitor) expression, and on cell cycle kinetics, were also examined. Treatment of ovarian cancer cells with carboplatin results in increased HuR cytoplasmic expression and elevated WEE1 expression, arresting cell cycle G2/M transition. This may explain why HuR cytoplasmic localization in chemo-naïve tumors is not predictive of therapeutic response and PFS following second-line gemcitabine/platin combination therapy. These results suggest treatment of recurrent ovarian tumors with a combination of gemcitabine, carboplatin, and a WEE1 inhibitor may be potentially advantageous as compared to current clinical practices.

## INTRODUCTION

Nearly 80% of ovarian cancer patients will have a favorable response to first-line therapy consisting of optimal surgical debulking followed by aggressive chemotherapy with paclitaxel and a platinum-based therapy [1]. Unfortunately, due to the development of chemoresistant disease, a majority of patients will develop recurrent tumors within 16-22 months. Gemcitabine in combination with carboplatin is commonly used as a second-line chemotherapy to treat recurrent disease. Some tumors respond better to a combination of gemcitabine and carboplatin than others, resulting in longer progression free survival (PFS). Unfortunately, the field does not have a reliable biomarker to determine which patients will respond well. Even when patients respond to the most aggressive therapy, almost all will eventually succumb to their disease.

Gemcitabine acts as a prodrug that, when metabolized to gemcitabine di- and tri-phosphates, functions to inhibit DNA elongation, DNA repair enzymes, and RNA synthesis [2, 3]. Potential clinical relevance of an association between the amount of cytoplasmic localization of an RNA-binding protein, HuR, in tumor cells and the metabolic activation of gemcitabine has recently been identified [4, 5]. HuR functions in normal, healthy cells as a critical molecule involved in post-transcriptional gene regulation. When cells are stressed, e.g., by low oxygen levels, HuR potently influences translation of key survival and growth-related mRNAs in the cytoplasm by several mechanisms including active transport of mRNAs from the nucleus to the cytoplasm, mRNA stabilization, and direct facilitation of translation. In line with these functions, HuR has been identified as a marker for poor prognosis in many cancers, including ovarian cancer [6–8]. In two small cohorts of pancreatic cancer patients treated with gemcitabine following surgery, a significant association between increased overall survival and high HuR expression in the cytoplasm has been identified, suggesting that HuR subcellular localization might serve as a predictive marker for gemcitabine response [4, 5]. Enhanced gemcitabine functionality in tumors is likely the consequence of increased production of the key nucleoside analog metabolizing enzyme, deoxycytidine kinase (dCK) resulting from post-transcriptional regulation of dCK mRNA by HuR [4, 5, 9, 10]. In this study, we sought, but found no evidence, that HuR cellular localization might serve as a predictive marker for clinical outcome following gemcitabine treatment of recurrent ovarian tumors. We explored potential reasons why this might be the case. The results of our findings suggest that improved clinical outcomes for ovarian cancer patients might be better achieved using gemcitabine in combination with carboplatin and a WEE1 inhibitor as a second-line therapy.

## RESULTS AND DISCUSSION

### Is HuR cellular localization in ovarian tumors predictive of favorable tumor response to gemcitabine?

**Figure 1:**
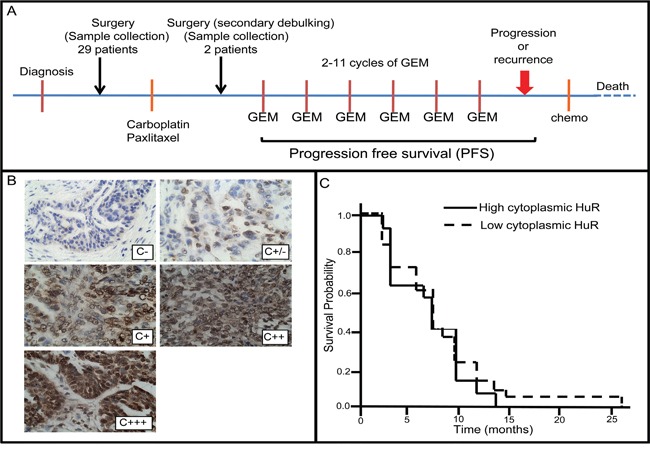
Analysis of HuR subcellular localization in ovarian tumor specimens and corresponding progression free survival of patients **A.** Treatment protocol for ovarian cancer patients with recurrent tumors. **B.** Ovarian tumor sections immunostained for HuR and representative of how staining intensity was scored. **C.** Kaplan-Meier plot of progression free survival (PFS) of patients with tumors having relatively low HuR cytoplasmic staining (scored -, +/−, or +) and tumors having high cytoplasmic HuR staining (scored ++ or +++).

We investigated whether there is a correlation between subcellular localization of HuR in tumor cells and PFS of ovarian cancer patients receiving gemcitabine as a second-line therapy for recurrent tumors. Sections of thirty-one ovarian tumor specimens from patients treated with gemcitabine were immunostained for HuR. Twenty-nine of the specimens were from chemo-naive tumors (i.e., surgically-debulked tumors prior to any chemotherapy), while two were from secondary surgically-debulked tumors following first-line chemotherapy with carboplatin and paclitaxel (Figure 1A). Twenty-nine of the 31 tumor specimens were from patients receiving second-line therapy with gemcitabine in combination with one or more chemotherapeutics – 26 with carboplatin, 1 with carboplatin and avastin, and 2 with cisplatin (Table 1). Two of the 31 tumors had HuR expression in only a few nuclei and no cytoplasmic HuR, 1/30 had only nuclear staining, while HuR was present in at least 50% of nuclei and in the cytoplasm of 28/30 tumors. The amount of cytoplasmic HuR in these 28 tumors varied: 7 were scored +/−, 9 were +, 6 were ++, and 6 were +++ (Figure 1B). To assess whether the amount of cytoplasmic HuR expression in tumors was predictive of PFS following gemcitabine/carboplatin, we compared PFS of patients whose tumors had low cytoplasmic HuR expression (−, +/−, +; n=19; median = 8 mo) with that of patients whose tumors had high cytoplasmic HuR expression (++ and +++; n=12; median = 8 mo) (Figure 1C). There was no significant difference in PFS (p=0.58). Even when tumors that were scored as + were grouped with ++ and +++ tumors, there was no significant difference in PFS (− and +/−; n=9; median = 8 mo) and (+, ++, +++; n=22; median = 8 mo) (p=0.62). The two tumor specimens that were collected after first-line therapy had cytoplasmic HuR scores of + and +++, and had PFS values of 3 mo and 4 mo, respectively. The PFS of the 3 patients whose tumors had no detectable HuR in the cytoplasm was near or above the median value (8 mo, 8 mo, 12 mo). These data provide no evidence to support the use of HuR subcellular localization as a predictive marker for ovarian tumor sensitivity to second line treatment with gemcitabine. We note, however, a correlation of lower tumor grades (Grades I and II) with low cytoplasmic HuR, and Grade III tumors with high cytoplasmic HuR approached significance (p=0.066) (Figure S1).

**Table 1:** Profile of patients in this study

Age (yrs)	61.8 +/− 9.3^*^
Race	Caucasian (29)
	African American (2)
Stage	Ib (1)
	Ic (1)
	II (1)
	IIb (1)
	IIc (1)
	IIIb (1)
	IIIc (24)
	unstaged (1)
Grade	g2 (4)
	g2-3 (3)
	g3 (24)
Histology	serous (27)
	serous/endometrioid (1)
	serous w/mucinous features (1)
	carcinosarcoma + serous (1)
	serous w/clear cell features (1)
Optimal debulking	20 optimal
	11 sub-optimal
# first-line therapy carboplatin + paclitaxel cycles	1.8 +/− 1.1^*^ (range: 1-5)
# second-line gemcitabine cycles (i.e., treatment of recurrent tumors)	5.1 +/− 1.9^*^ (range: 3-11)
Second-line gemcitabine therapy combined with other chemo?	Carboplatin (26)
	Carboplatin + avastin (1)
	Cisplatin (2)
	None (2)
Progression Free Survival (mo)	8.5 +/− 4.8^*^ (range: 3-26)

* Mean +/− S.D.

Patients in this study were treated with different numbers of cycles of carboplatin/paclitaxel (1X, n=18; 2X, n=7; 3X, n=2; 4X, n=3; 5X, n=5), thereby contributing variability to the duration of first-line carboplatin/paclitaxel therapy and the time between assessment of HuR localization status in surgically-debulked tumors and the initiation of gemcitabine treatment. In addition, patients were treated with different numbers of cycles of gemcitabine as second-line therapy (range 2-11), with most patients treated 5 (n=8) or 6 (n=11) times, and with gemcitabine in combination with different chemotherapeutic drugs. These variables may contribute to our failure to identify a correlation between HuR cellular localization in ovarian tumors and PFS as was observed in pancreatic adenocarcinoma where gemcitabine is administered to patients as a first-line therapy. We investigated possible molecular mechanisms that might be operative in the context of these variables.

### dCK activates gemcitabine in ovarian cancer cells

Deoxycytidine kinase (dCK) is the rate limiting metablic enzyme that phosphorylates deoxyribonucleosides as well as their nucleoside analogs (e.g., gemcitabine) [11]. To confirm that dCK activates gemcitabine in ovarian cancer cells, as has been demonstrated in pancreatic cancer cells [4], we transiently transfected A2780 cells with siRNA against dCK (siDCK) or siControl (siCtrl) for 24 h, then treated cells with various concentrations of gemcitabine for 48 h and measured viable cells. In parallel cultures, we confirmed dCK suppression in siDCK-transfected cells by western blot analysis (Figure S2). dCK inhibition resulted in ~80% increase in cell number when cells were treated with 0.005 and 0.01 μM gemcitabine as compared to gemcitabine-treated siCtrl-transfected and untransfected cells (p<0.005) (Figure S2). These results support gemcitabine activation by dCK in ovarian cancer cells.

### HuR binds to dCK mRNA in ovarian cancer cells

**Figure 2:**
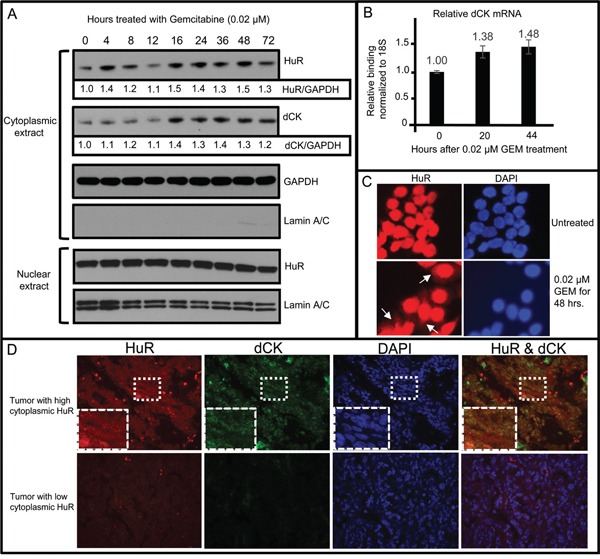
HuR nuclear to cytoplasmic translocation following treatment of OVCAR5 cells with gemcitabine (GEM) associated with an increase in cytoplasmic dCK mRNA and protein **A.** Western blot analysis for HuR and dCK in cytoplasmic and nuclear protein lysates. GAPDH provided loading control and allowed for quantitative comparison of HuR and dCK at different time points (values indicated beneath HuR and dCK panels). Lamin A/C provides marker for cytoplasmic extract purity. **B.** qRT-PCR analysis of cytoplasmic dCK mRNA isolated from OVCAR5 cells treated with GEM for different times (mean +/− SD). **C.** OVCAR5 cells grown in medium +/− GEM and immunostained for HuR. White arrows point to cytoplasmic HuR. **D.** Sections of human ovarian tumor, collected prior to drug treatment, were immunostained for HuR and dCK. Boxed area is enlarged in lower left corner of each panel in top row.

HuR protein binds to specific mRNA transcripts in a highly regulated process subject to modifications of HuR protein itself (e.g., phosphorylation and methylation) and to competition with miRNAs for binding sites [12–16]. We have shown that, upon gemcitabine treatment, HuRbinds to dCK mRNA in pancreatic tumor cells, resulting in increased dCK protein and activity [4]. In addition, we have shown a strong correlation between dCK and HuR cytoplasmic expression in pancreatic ductal adenocarcinomas [9]. Our recent studies identified specific mRNAs bound to HuR in ovarian and pancreatic cancer cells that are unique to these two cancer types [17, 18]. It is therefore possible that HuR may not directly associate with dCK mRNA in ovarian cancer cells as it does in pancreatic cancer cells, thereby offering a possible explanation why HuR cytoplasmic localization failed to serve as an informative marker for gemcitabine sensitivity. To test whether HuR translocation from the nucleus to the cytoplasm in response to gemcitabine correlates with an increase in dCK expression in ovarian cancer cells, we treated OVCAR5 cells with 0.02 μM gemcitabine (IC_50_), prepared cytoplasmic and nuclear protein fractions at various time points, and analyzed the amount of HuR and dCK proteins in these two cellular compartments on western blots. Cytoplasmic HuR and dCK levels peaked between 16 h and 48 h after gemcitabine treatment (~1.5-fold higher than level at 0 h) (Figure 2A). An increase in dCK mRNA was also observed by qRT-PCR during this timeframe (Figure 2B). Increased cytoplasmic HuR at 48 h after gemcitabine treatment was also observed by immunofluorescent staining (Figure 2C). The amount of nuclear HuR, however, appeared unchanged upon gemcitabine treatment (Figure 2A), most likely due to masking of subtle changes in its abundance that are not detected on western blots. The same analysis of another ovarian cancer cell line, A2780, confirmed the association of gemcitabine-induced elevation of cytoplasmic HuR and dCK mRNA/protein (Figure S3). Sections of human ovarian tumors immunostained for HuR and dCK revealed a positive correlation (p=0.008) between cytoplasmic HuR expression and dCK expression (Figure 2D). These results show that gemcitabine treatment induces HuR translocation to the cytoplasm and that this translocation is associated with increased dCK expression in ovarian cancer cells.

**Figure 3:**
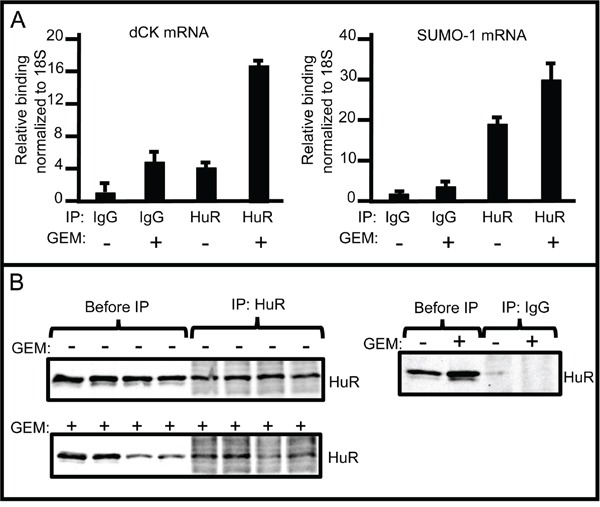
RNP-IP assays showing increased binding of dCK mRNA to HuR in response to gemcitabine **A.** HuR protein-bound dCK (left) and SUMO-1 (right) mRNA amounts in A2780 cells grown in the presence or absence of 1μM GEM for 12 h as measured by qPCR (mean +/− SD). **B.** Left: Western blots of protein lysates prepared from A2780 cells grown in the presence or absence of 1 μM GEM, before immunoprecipitation, and immunoprecipitates prepared with anti-HuR. Three separate IPs were assayed. Right: Western blots of protein lysates before immunoprecipitation, and immunoprecipitates prepared with IgG.

The dCK 3′UTR region contains 8 putative HuR recognition motifs [19]. To determine whether HuR binds directly to dCK mRNA in ovarian cancer cells, we performed RNP-IP assays on lysates prepared from A2780 cells grown in the presence or absence of gemcitabine for 12 hours. An RNP-IP with a HuR-specific antibody was performed to isolate total mRNA transcripts associated with HuR, followed by qRT-PCR to determine the amount of dCK mRNA associated with HuR protein. IgG RNP-IP was performed as a negative control (Figure 3). The dCK mRNA level was increased 4-fold in gemcitabine-treated cells compared to untreated cells. SUMO-1 mRNA, a HuR target (unpublished, Brody Lab), served as a positive control and was enriched ~2 fold upon gemcitabine stress (Figure 3). The same study performed in OVCAR3 cells confirmed the observation that dCK mRNA isolated from HuR RNP-IP samples were significantly enriched (Figure S4). These results indicate that HuR binds directly to dCK mRNA in ovarian cancer cells, just as it does in pancreatic cancer cells.

### HuR suppression reduces dCK expression and gemcitabine efficacy

**Figure 4:**
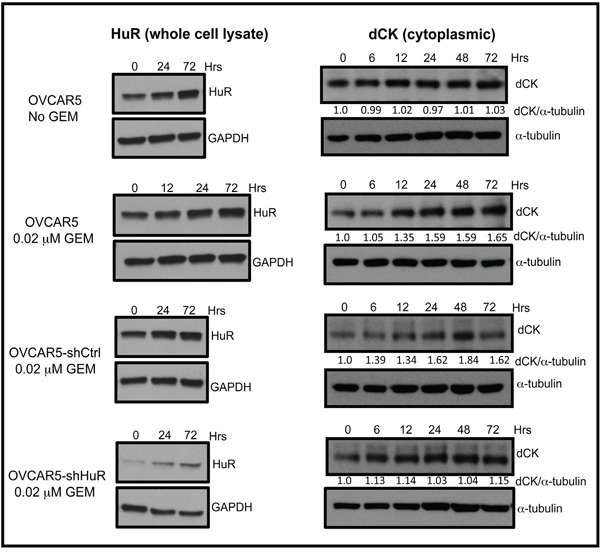
HuR suppression inhibits increase in dCK expression in response to GEM Western blot analysis of HuR and dCK protein expression in OVCAR5, OVCAR5-shCtrl, and OVCAR5-shHuR cells treated with or without 0.02 μM gemcitabine (GEM) for indicated times.

To study HuR regulation of dCK further, we examined the effect of HuR silencing on dCK expression. We generated an ovarian cancer cell line, OVCAR5-shHuR_c_257 that stably expresses short hairpin RNA (shHuR) [17]. We stressed OVCAR5, OVCAR5-shCtrl, and OVCAR5-shHuR_c_257 cells with 0.02 μM gemcitabine for 0, 6, 12, 24, 48, and 72 h, then prepared whole cell lysates to measure dCK expression on western blots (Figure 4). dCK protein expression in gemcitabine-treated OVCAR5 and OVCAR5-shCtrl cells increased (35-84%) in a time dependent manner (Figure 4). In contrast, dCK expression in gemcitabine-treated OVCAR5-shHuR_c_257 cells remained unchanged at all time points except 72 h when a minor increase was observed (Figure 4). A small increase in total HuR expression was observed in gemcitabine-treated OVCAR5-shHuR_c_257 cells. This observation likely reflects previous reports of HuR self-regulation [20–22].

**Figure 5:**
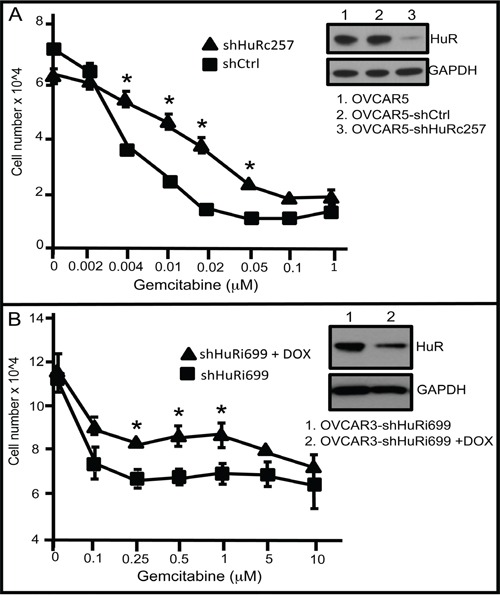
HuR suppression reduces gemcitabine chemotherapeutic efficacy **A.** Number of OVCAR5-shCtrl or OVCAR5-shHuR cells at 72 h following treatment with different concentrations of GEM. Western blot analysis of HuR of whole cell lysates in OVCAR5-shCtrl or OVCAR5-shHuR cells (mean +/− SD). **B.** Numbers of OVCAR3-shHuRi699 cells treated with +/− 0.1μg/ml DOX and with different GEM concentrations. Western blot analysis of HuR in OVCAR3-shHuRi699 cultured in GEM-containing medium with or without DOX (mean +/− SD). shHuRi = inducible shHuR; shHuRc = constitutively expressed shHuR. *indicates p<0.05

Next, we determined the impact of HuR inhibition on ovarian cancer cell sensitivity to gemcitabine. We treated two cell lines, OVCAR5-shHuR_c_257 and OVCAR3-shHuR_i_699, a doxycycline (DOX)-inducible HuR-targeted shRNA ovarian cancer cell line, with various gemcitabine concentrations for 72 h, and then assessed cell viability. HuR suppression in OVCAR5-shHuR_c_257 and DOX-induced OVCAR3-shHuR_i_699 cells compared to control cells resulted in reduced sensitivity to gemcitabine (Figure 5). In sum, these experiments show that HuR translocation to the cytoplasm in response to gemcitabine exposure results in increased dCK expression. This, in turn, results in enhanced gemcitabine therapeutic efficacy in ovarian cancer cells. It is clear that there must be another confounding factor or factors that compromise the value of HuR cellular localization as a predictive marker of second-line gemcitabine efficacy.

### Combination chemotherapy abrogates HuR's ability to act as a predictive marker for gemcitabine efficacy

Nearly all of the tumor samples examined in our patient study (29/31) were obtained from surgical debulking procedures performed prior to first-line carboplatin/paclitaxel treatment. Given our previous study in which we showed HuR translocation from the nucleus to the cytoplasm in pancreatic cancer cells upon stress with DNA-damaging anticancer agents including carboplatin and paclitaxel [23], it is reasonable to expect an increase in cytoplasmic HuR in response to exposure to first-line treatment, far in advance of gemcitabine/carboplatin second-line therapy. The fact that 12/29 of the tumors had significant cytoplasmic HuR prior to first-line therapy, and the PFS of these patients was not significantly different from that of patients with low cytoplasmic HuR, is further evidence that the complexity of HuR biology abrogates its use as a predictive marker for efficacy of gemcitabine second-line therapy. Ideally, it would be preferable to determine HuR status just prior to gemcitabine therapy, but surgical debulking of recurrent tumors is rarely performed, limiting the clinical feasibility of attaining tumor specimens.

**Figure 6:**
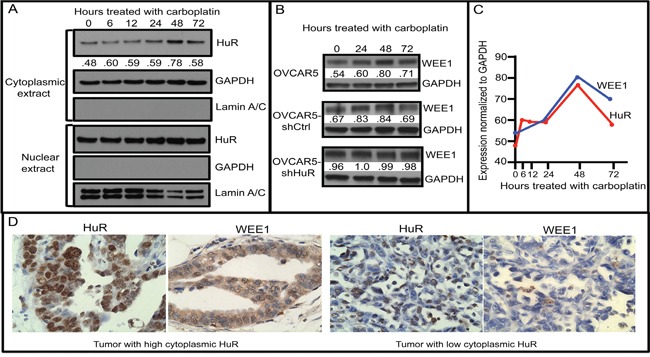
Cytoplasmic HuR increase in response to carboplatin increases WEE1 expression **A.** Western blot analysis of HuR and WEE1 proteins in OVCAR5 cells treated with 7.5 μM carboplatin for various times (0-72 h). Cytoplasmic and nuclear extracts were analyzed separately for HuR expression. GAPDH (a cytoplasmic marker) and Lamin A/C (a nuclear marker) analysis was also done to evaluate purity of cytoplasmic and nuclear extract preparations. **B.** WEE1 expression in OVCAR5 cells stably transfected with shHuR or with shCtrl (whole cell extracts). **C.** Cytoplasmic HuR and WEE1 expression in OVCAR5 cells treated with carboplatin for various times. Numbers in panels A and B indicate expression levels of HuR and WEE1 normalized to GAPDH. **D.** Sections of human ovarian tumor, with high (left) and low (right) HuR and WEE1 expression collected prior to drug treatment, immunostained for HuR and WEE1.

Another complication is that gemcitabine is nearly always administered to ovarian cancer patients in combination with one or more chemotherapeutics, usually carboplatin. A recent study showed that DNA-damaging radiation therapy, given in combination with gemcitabine, may disrupt HuR's ability to act as an informative biomarker (9). Expression of WEE1, a mitotic inhibitor kinase that regulates the DNA damage repair pathway, is present in ovarian cancer ascites following chemotherapy [24]. We have shown that an increase in HuR in response to DNA-damaging chemotherapeutics (e.g., carboplatin) results in elevated WEE1 in pancreatic cancer cells [23]. Elevated Wee1, in turn, promotes cell-cycle arrest at the G2/M transition and ensuing resistance to DNA-damaging agents. To explore whether HuR regulation of WEE1 provides a mechanism underlying the lack of improved PFS associated with cytoplasmic HuR in ovarian tumors of patients treated with a combination of gemcitabine and carboplatin, we first assayed HuR expression in OVCAR5 cells treated with 7.5 μM carboplatin (IC_50_) for various times. Cytoplasmic HuR began to increase after 24 h treatment with carboplatin and reached a maximum increase of 1.6-fold higher than in non-treated cells at 48 h (Figure 6A). The amount of nuclear HuR remained high in treated cells at all time points. WEE1 expression also increased in carboplatin-treated OVCAR5 cells, reaching a peak expression at 48 h, similar to HuR, whereas no increase in WEE1 was observed in carboplatin-treated OVCAR5-shHuR cells in which HuR expression was significantly inhibited (Figures 6A-6D). WEE1 protein increased 1.3-fold in HuR-expressing OVCAR5-shCtrl cells upon carboplatin treatment (Figure 6B & 6C).

We also determined the effect of HuR-regulated WEE1 expression on cell-cycle kinetics in the context of carboplatin treatment (Figures S5A & B). We observed that a higher percentage of cells accumulated in the G2/M phase in OVCAR5-shCtrl cells (29.5%) as compared to OVCAR5-shHuR (22.3%) 48 h after 7.5 μM carboplatin treatment (Figure S5B). Under normal culture conditions, the percentage of OVCAR5-shCtrl and OVCAR5-shHuR cells in the G2/M phase is similar (Figure S5B). In agreement with these *in vitro* observations, human ovarian tumor sections immunostained for HuR and WEE1 revealed a positive correlation between cytoplasmic HuR expression and WEE1 expression (p=0.048) (Figure 6D). These results offer a mechanism to explain why cytoplasmic localization of HuR is not predictive of a favorable outcome to gemcitabine treatment in our study when given as a combination therapy with carboplatin.

**Figure 7:**
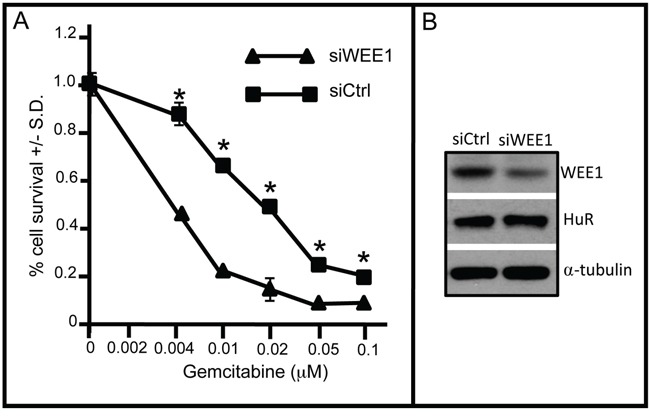
WEE1 inhibition sensitizes OVCAR5 cells to gemcitabine **A.** OVCAR5 cells were transfected with siWEE1 or siCtrl for 6 h. Following addition of gemcitabine at various concentrations to the culture medium, cell viability was assayed after 72 h. **B.** Western blot of WEE1 in gemcitabine-treated OVCAR5 cells treated with siHuR or siCtrl. α-tubulin serves as a gel loading control. *indicates p<0.0001

Since arrest of DNA replication by insertion of the gemcitabine analogue metabolite, triphosphate cytosine, is dependent on cell division, its effectiveness is likely to be compromised to some degree in cell cycle-arrested carboplatin-treated cells even though dCK metabolizes gemcitabine as a consequence of elevated HuR cytoplasmic expression. Clinical experience clearly shows, however, that in ovarian cancer patients with platinum-sensitive relapse, progression-free survival is prolonged when gemcitabine is given in combination with carboplatin as compared to carboplatin monotherapy [25]. Evidence suggests that this synergy may result from the inhibition of repair of platinum-induced DNA cross-links by gemcitabine [26, 27]. Our results suggest that patients with recurrent tumors be treated first with gemcitabine followed by treatment with carboplatin. To test directly the effect of WEE1-mediated cell cycle arrest on gemcitabine efficacy, we measured survival of OVCAR5 cells grown in medium containing various concentrations of gemcitabine in the presence or absence of siWEE1. WEE1 inhibition increased the sensitivity of cells to gemcitabine 2-4 fold over the range of tested gemcitabine concentrations, and decreased the IC_50_ from 0.02 to 0.004 μM (Figure 7). This result suggests that it may also be advantageous to combine inhibition of WEE1 with gemcitabine andcarboplatin as a combination second-line therapy, thereby overcoming cell-cycle arrest and enhancing the therapeutic response to gemcitabine in patients with platinum-sensitive relapse. A small molecule WEE1 inhibitor, MK-1775, has been shown to enhance antitumor efficacy of p53-deficient tumor cells to DNA-damaging agents including cisplatin, carboplatin, gemcitabine and 5-fluorouracil [28–30], and a Phase II clinical trial (NCT02101775) testing MK-1775 in combination with gemcitabine to treat recurrent ovarian cancer is currently recruiting. Given our understanding of how gemcitabine affects tumor cell survival, addition of gemcitabine to this therapeutic strategy may have added benefit to all patients independent of p53 status.

One limitation of our study is that HuR localization was analyzed in only one ovarian cancer subtype, serous ovarian tumors, a large majority of which were high-grade tumors. While this subtype accounts for ~70% of ovarian tumors, these tumors differ from other tumor subtypes (endometrial, clear cell, mucinous) not only in morphology but also in gene expression profile, molecular genetic features, genetic and epidemiologic risk factors, precursor lesions, pattern of spread, and of particular relevance to this study, response to platinum-taxane based treatment [31, 32]. Indeed, expression of hENT1, dCK, 5′NT, and RRM1 was found to be higher in undifferentiated and clear cell carcinoma as compared to serous ovarian tumors [33]. Given these substantial differences, the possibility that HuR localization might be an informative marker for gemcitabine response in other ovarian tumor subtypes warrants further study.

## MATERIALS AND METHODS

### Patient population

Medical records from our gynecologic oncology practice (CJD) at the Lankenau Medical Center, Wynnewood, PA, were reviewed to identify ovarian cancer patients who had undergone surgical debulking and first-line treatment with carboplatin and paclitaxel, who subsequently developed recurrent tumors and underwent further therapy with gemcitabine in combination with carboplatin (26/31) or other chemotherapeutic (3/31). Using patients' medical record numbers to assure sample de-identification, archival paraffin blocks of tumor tissue from 31 patients were retrieved and sections were prepared. This protocol was approved by the Lankenau Medical Center Institutional Review Board. The patient profile is summarized in Table 1. Progression free survival time was determined by CA125 Resist based on serial CA125 measurements, as well as on CT scans.

### Generation of HuR knock-down cell lines

HuR stable knock-down cell lines were generated in our lab as previously described [17]. Briefly, to generate OVCAR5 cells stably-expressing shHuR_c_257 (“c” for constitutive) or shCtrl, cells were infected with shHuR_c_257- or shCtrl-expressing lentivirus. Twenty-four hours after viral infection, shHuRc257- or shCtrl-expressing cells were selected in medium containing hygromycin B (Gemini #400-123). To generate OVCAR3 Dox-inducible shHuR_i_699 cells (“i” for inducible), cells were infected with shHuRi699-expressing lentivirus. Forty-eight hours post-viral infection, shHuR- or shCtrl-expressing cells were selected in medium containing puromycin dihydrochloride (Gemini # 400-128P).

### Cell Culture, transfection, chemo-treatment, and viability assay

OVCAR5 cells (A. Klein-Szanto, Fox Chase Cancer Center) and A2780 cells (T. Hamilton, Fox Chase Cancer Center) were maintained in RPMI-1640 medium (Cellgro) supplemented with 10% FBS (Gemini) at 37°C in 5% CO_2_. OVCAR3 cells were purchased from American Type Culture Collection (ATCC) and grown in media as recommended by ATCC. All cells tested negative for mycoplasma using a PCR kit (Sigma #MP0035-1KT). For in vitro transfection, cells were transiently transfected with siDCK (Ambion #4390824), siWEE1 (Thomas Scientific Dharma #GLEKC-000008), or siControl (Ambion #AM4611) using Lipofectamine 2000 (Invitrogen) according to the manufacturer's directions. To determine effects of gemcitabine on HuR and dCK expression, or carboplatin on HuR and WEE1 expression, cells were incubated in IC_50_ concentrations of gemcitabine (0.02 μM) or in carboplatin (7.5 μM) for various times, then harvested for Western blot analysis. To determine effects of gemcitabine on HuR knockdown cell proliferation, OVCAR5-shHuR_c_257 cells were incubated with various concentrations of gemcitabine for 72 hours and OVCAR3-shHuR_i_699 cells were incubated with 1μg/ml doxycycline and various concentrations of gemcitabine for 72 hours, after which viable cell number was determined using EZ Count Kit (Rockland #KLD-001). All cell viability assays were done in triplicate.

### Immunoblot analysis

To prepare whole cell extracts, cells were homogenized in RIPA buffer containing proteinase inhibitors and centrifuged at 12,000 rpm for 15 min at 4°C. Cytoplasmic and nuclear cell extracts were prepared [Nuclear and Cytoplasmic Extraction kit (Pierce #78833)] and protein concentration of extracts was determined [BCA Protein Assay Kit (Pierce #23225)]. Soluble proteins were separated on 10% SDS-PAGE gels and analyzed by Western blotting using antibodies toHuR (1:1000) (clone 3A2; Santa Cruz Biotechnology), dCK (1:500) (clone 2243C2; Santa Cruz), WEE1 (1:1000) (clone B11; Santa Cruz), lamin A/C (1:1000) (clone 636; Santa Cruz Biotechnology), or GAPDH (1:8000) (clone 5C6; Ambion). Blots were washed several times with PBST (phosphate buffered saline, 0.1% Tween), then incubated with horseradish peroxidase-labeled goat anti-mouse (1:8000) (Thermo Scientific) secondary antibody. Proteins were visualized with ECL (Pierce #32106). Densitometry quantification of proteins was done using Image J software.

### Immunostaining

Antigen retrieval was performed on deparaffinized tumor sections by steam heating for 30 min in citrate buffer followed by endogenous peroxidase quenching with 3% H_2_O_2_/methanol for 20 min. Cells were grown in chamber slides, then fixed in cold acetone at −20°C for 10 minutes. Tumor sections or cells were incubated with primary antibody and biotinylated secondary antibody. Signals were amplified and visualized either using the TSA-Plus Fluorescence System (Perkin Elmer) according to manufacturer's instructions, or using avidin/biotin complex system (VECTASTAIN Elite ABC kit from VECTOR Laboratories) followed by DAB visualization and hematoxylin counterstaining. Slides were imaged with a Zeiss Axiovert 200M microscope or Zeiss Axioplan microscope. Human ovarian tumor HuR cytoplasmic staining was scored as -, +/−, ++, or +++. Stained sections were viewed and scored twice by two different pairs of investigators (JAS and WP; JAS and GSD) who were blind to PFS of each patient. Anti-HuR monoclonal antibody 19F12 (1:5000) was from Clonegene. Anti-dCK (Abcam #ab151966) was from Abcam. Anti-WEE1 (clone B-11) was from Santa Cruz.

### Ribonucleoprotein-immunoprecipitation (RNP-IP)

RNP-IPs were performed as described [34]. Briefly, A2780 or OVCAR3 cells were treated with 1μg/ml gemcitabine for 12 hours, then cytoplasmic lysates were obtained using CelLytic NuCLEAR Extraction Kit (Sigma-Aldrich, #NXTRACT) according to manufacturer's instructions, with the modification of supplementing with 100 U/ml RNase inhibitor (Life Technologies, # N8080119) to preserve RNA integrity. HuR protein and its bound mRNA cargo were immunoprecipitated by incubating the cytoplasmic lysates with mRNP-IP-grade HuR antibody (MBL International Corp. #RN004P) or isotype control IgG (Santa Cruz #sc-2027) pre-coated to Protein A-Sepharose beads (Sigma-Aldrich #P9424). HuR was digested with proteinase K (Life Technologies, cat. #AM2546). After IP, the RNA was isolated and cDNA synthesized (A&B applied biosystems #4368814). GAPDH and dCK transcripts were quantified by real-time PCR analysis (#4352024) using specific probes, dCK (#Hs01040726_m), 18S (#Hs99999901_s1), and SUMO-1(#Hs02339312_g1) (A&B Applied Biosystems). The relative levels of dCK product were first normalized to 18S product in all IP samples, then fold changes in HuR-IP were compared with IgG-IP.

### Statistical analysis

The distribution of PFS was estimated using the Kaplan-Meier method and association of HuR expression with PFS was assessed using the log-rank test. Correlations of tumor grade with HuR expression, HuR and dCK expression, and HuR and WEE1 expression were evaluated using Fisher's exact test.

## SUPPLEMENTARY FIGURES


