# Supplementary material for: Insights from HuR biology point to potential improvement for second-line ovarian cancer therapy
Source: Oncotarget. 2016 Mar 2;7(16):21812–24. doi: 10.18632/oncotarget.7840 (PMC5008325; doi:10.18632/oncotarget.7840)
Supplement: Supplementary file 1 [file oncotarget-07-21812-s001.pdf]

SUPPLEMENTARY FIGURES

Cytoplasmic HuR status

Tumor grade		+/-, +	++/+++
	1-2	5	0
	3	13	12

* 1 ungraded tumor (+)

Supplementary Figure S1: Correlation of cytoplasmic HuR status and tumor grade. (n = 30; one tumor in the study was ungraded. **A.** correlation approached significance ($p = 0.066$) by Fisher's exact test.

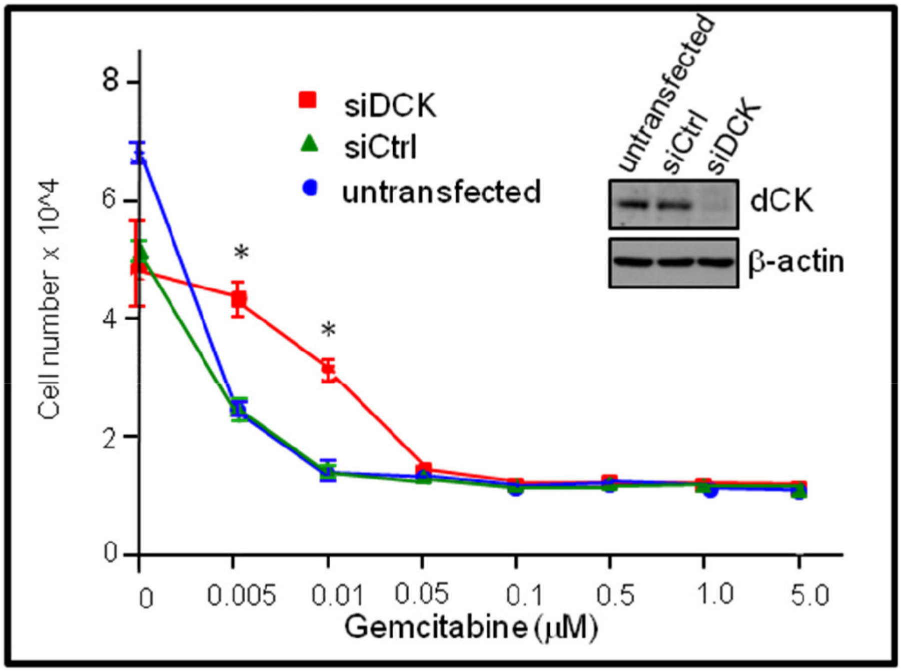

Supplementary Figure S2: dCK silencing in ovarian cancer cells reduces gemcitabine response. Number of A2780, siCtrl-, and siHuR- transfected A2780 cells treated with gemcitabine at the indicated concentrations. * indicates p<0.005

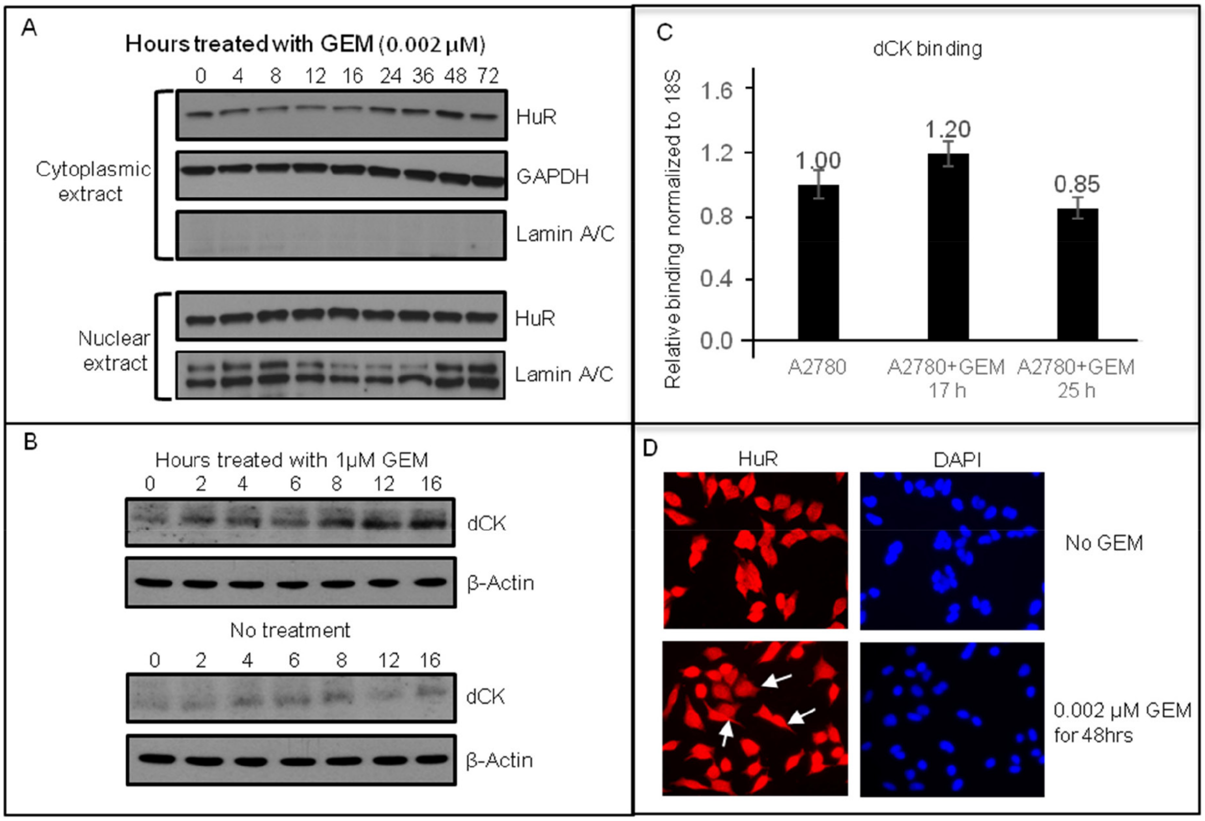

Supplementary Figure S3: HuR translocation from the nucleus to the cytoplasm dCK expression following treatment of A2780 cells with gemcitabine for different times. A. Western blot analysis of HuR in cytoplasmic and nuclear protein lysates. Lamin A/C provides marker for cytoplasmic extract purity. **B.** Western blot analyses of dCK in whole cell protein lysates. **C.** qRT-PCR analysis of cytoplasmic dCK mRNA isolated from A2780 cells treated with gemcitabine for different times. **D.** A2780 cells grown in medium +/- gemcitabine and immunostained for HuR. Arrows point to examples of cytoplasmic staining

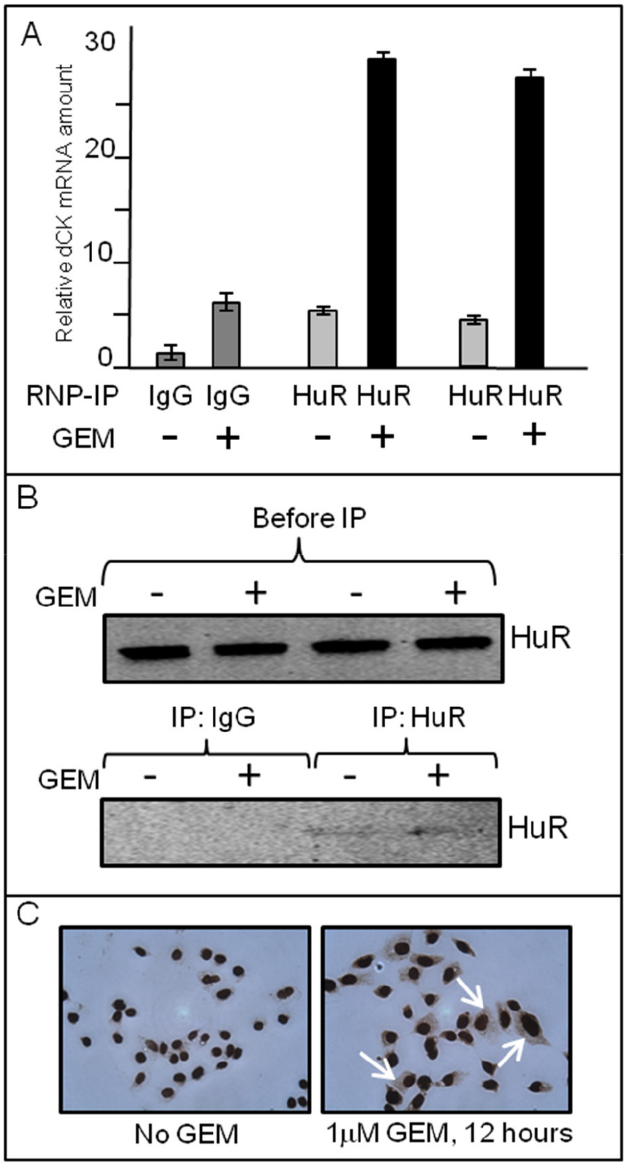

Supplementary Figure S4: HuR response to gemcitabine regulates dCK expression. A. RNP-IP assays showing amount of HuR protein-bound dCK mRNA in the presence and absence of GEM as measured by qPCR. Two independent sets of cell lysates were assayed. **B.** Western blots of protein lysates prepared from OVCAR3 cells that were grown in the presence or absence of 1 mM GEM before immunoprecipitation (top). Western blot of immunoprecipitates prepared with IgG or anti-HuR is shown on the bottom. **C.** OVCAR3 cells grown in medium +/- GEM and immunostained for HuR. Arrows point to examples of cytoplasmic staining.

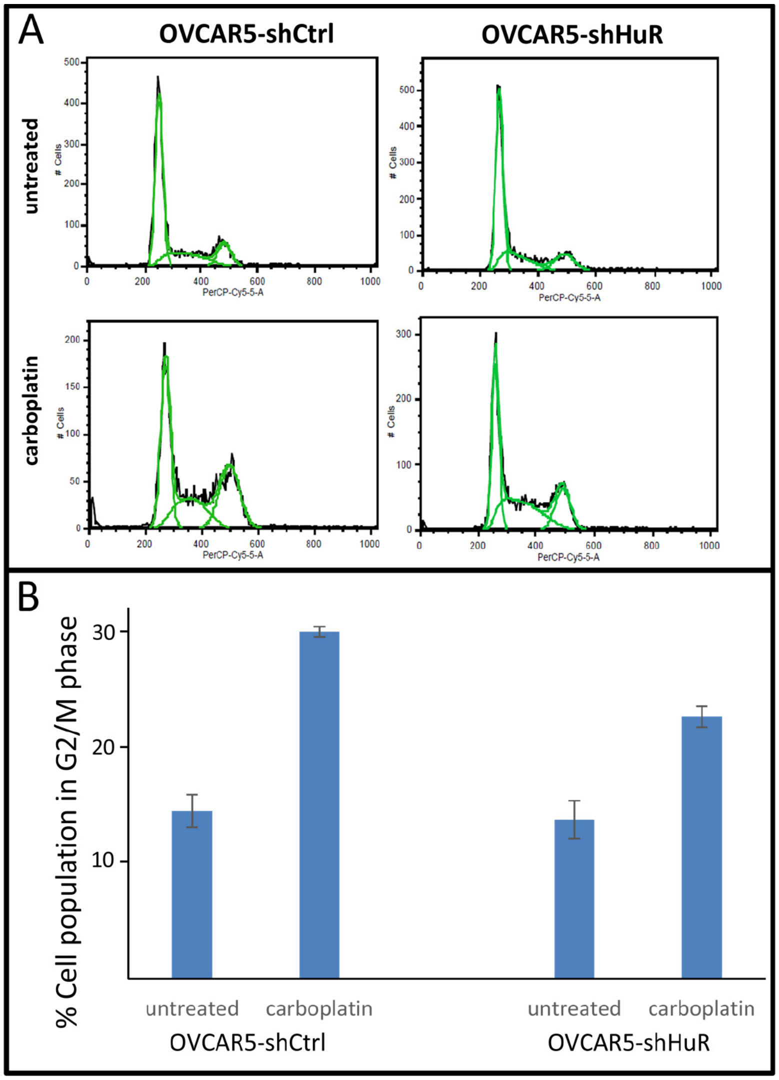

Supplementary Figure S5: HuR inhibition upon carboplatin treatment reduces accumulation of cells in G2/M phase. **A.** Cell-cycle kinetics in OVCAR5-shCtrl and OVCAR5-shHuR cells treated with or without 7.5 µM carboplatin in the G/M phase. **B.** Percentage of OVCAR5-shCtrl and OVCAR5-shHuR cells in the G2/M phase following treatment with or without 7.5 mM carboplatin.
